# Epidemiological characteristics of tuberculosis incidence and its macro-influence factors in Chinese mainland during 2014–2021

**DOI:** 10.1186/s40249-024-01203-6

**Published:** 2024-05-21

**Authors:** Le-le Deng, Fei Zhao, Zhuo-wei Li, Wei-wei Zhang, Guang-xue He, Xiang Ren

**Affiliations:** 1 National Institute for Viral Disease Control and Prevention, Chinese Center for Disease Control and Prevention, Beijing, 102206 China; 2 Department of Pharmacy, Beijing Hospital; National Center of Gerontology; Institute of Geriatric Medicine, Chinese Academy of Medical Science; Beijing Key Laboratory of Drug Clinical Risk and Personalized Medication Evaluation, Beijing, 100730 China; 3 Miyun District Center for Disease Control and Prevention, Beijing, 101500 China; 4 School Of Public Health, Binzhon Medical University, Yantai, 264000 China; 5 Division of Infectious Disease, Key Laboratory of Surveillance and Early-Warning On Infectious Disease, Chinese Center for Disease Control and Prevention, Beijing, 102206 China

**Keywords:** Tuberculosis, Surveillance, Epidemiological characteristics, Incidence, Influence factor, Distributed lag nonlinear model (DLNM), Spatial panel model, China

## Abstract

**Background:**

Tuberculosis (TB) remains a pressing public health issue, posing a significant threat to individuals' well-being and lives. This study delves into the TB incidence in Chinese mainland during 2014–2021, aiming to gain deeper insights into their epidemiological characteristics and explore macro-level factors to enhance control and prevention.

**Methods:**

TB incidence data in Chinese mainland from 2014 to 2021 were sourced from the National Notifiable Disease Reporting System (NNDRS). A two-stage distributed lag nonlinear model (DLNM) was constructed to evaluate the lag and non-linearity of daily average temperature (℃, Atemp), average relative humidity (%, ARH), average wind speed (m/s, AWS), sunshine duration (h, SD) and precipitation (mm, PRE) on the TB incidence. A spatial panel data model was used to assess the impact of demographic, medical and health resource, and economic factors on TB incidence.

**Results:**

A total of 6,587,439 TB cases were reported in Chinese mainland during 2014–2021, with an average annual incidence rate of 59.17/100,000. The TB incidence decreased from 67.05/100,000 in 2014 to 46.40/100,000 in 2021, notably declining from 2018 to 2021 (APC = -8.87%, 95% *CI*: -11.97, -6.85%). TB incidence rates were higher among males, farmers, and individuals aged 65 years and older. Spatiotemporal analysis revealed a significant cluster in Xinjiang, Qinghai, and Xizang from March 2017 to June 2019 (*RR* = 3.94, *P* < 0.001). From 2014 to 2021, the proportion of etiologically confirmed cases increased from 31.31% to 56.98%, and the time interval from TB onset to diagnosis shortened from 26 days (IQR: 10–56 days) to 19 days (IQR: 7–44 days). Specific meteorological conditions, including low temperature (< 16.69℃), high relative humidity (> 71.73%), low sunshine duration (< 6.18 h) increased the risk of TB incidence, while extreme low wind speed (< 2.79 m/s) decreased the risk. The spatial Durbin model showed positive associations between TB incidence rates and sex ratio (*β* = 1.98), number of beds in medical and health institutions per 10,000 population (*β* = 0.90), and total health expenses (*β* = 0.55). There were negative associations between TB incidence rates and population (*β* = -1.14), population density (*β* = -0.19), urbanization rate (*β* = -0.62), number of medical and health institutions (*β* = -0.23), and number of health technicians per 10,000 population (*β* = -0.70).

**Conclusions:**

Significant progress has been made in TB control and prevention in China, but challenges persist among some populations and areas. Varied relationships were observed between TB incidence and factors from meteorological, demographic, medical and health resource, and economic aspects. These findings underscore the importance of ongoing efforts to strengthen TB control and implement digital/intelligent surveillance for early risk detection and comprehensive interventions.

**Graphical Abstract:**

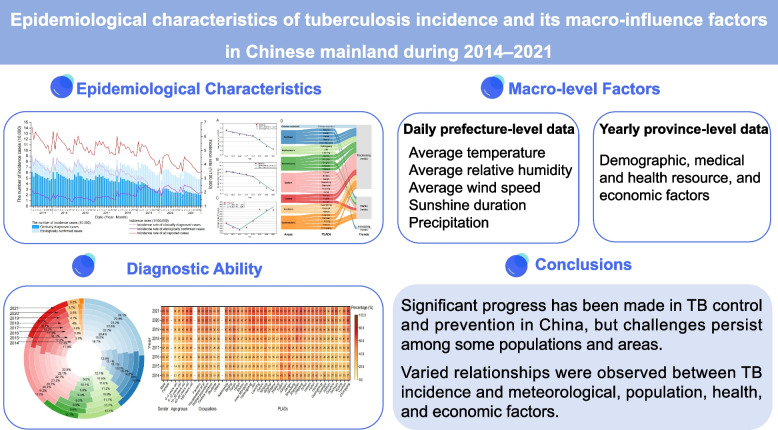

**Supplementary Information:**

The online version contains supplementary material available at 10.1186/s40249-024-01203-6.

## Background

Tuberculosis (TB) has long been a global public health challenge, with approximately 10.6 million cases reported worldwide in 2022 [1]. Despite advancements in medical technology and healthcare, TB remains the leading infectious cause of death, claiming 1.5 million lives annually [2]. According to the World Health Organization (WHO), eight countries, including China, accounted for two-thirds of global TB cases in 2022 [1]. China, the third-largest contributor to global TB burden [1], reported an incidence rate of 45.37/100,000 in 2021 [3]. While China has made commendable progress in TB control, ongoing attention is required for high-risk areas and populations [4]. Therefore, a comprehensive understanding of the demographic, temporal, and spatial distribution of TB is essential for effective interventions.

Although several studies have explored the epidemiological features of TB in China over different periods and regions [5–8], this study distinguishes itself through its detailed analysis and extended temporal scope. Utilizing data from the National Notifiable Disease Reporting System (NNDRS), this study provides a more thorough analysis of the demographic, temporal, and spatial aspects of TB incidence from 2014 to 2021.

Various macro-level factors, such as climate change, population migration, and urbanization, significantly influence infectious disease patterns [9]. Recent researches have investigated the relationship between these macroscopic factors and TB incidence, using diverse statistical analyses [4, 5, 10, 11]. The logistic and line regression models were usually used for exploring the linear relationship, based on the individual data. However, studies have proved the relationship between meteorological factors and TB incidence was nonlinear and lagged [10–12]. Based on the data in our study, the distributed lag nonlinear model (DLNM) can effectively capture the non-linear and lagged relationship between meteorological factors and TB incidence, revealing exposure-lag-response effects [10–12]. A study employing DLNM found temperature, relative humidity, and wind speed playing crucial roles in TB incidence with delayed and non-linear effects in Urumqi, China [10]. Additionally, spatial panel data models have been employed to analyze the relationship between socioeconomic factors and TB incidence, accounting for spatial dependence and heterogeneity [13, 14]. However, all these studies have been conducted in different geographic locations which focus on the relationship of someone factors with TB incidence. The comprehensive nationwide studies exploring the relationship between factors from meteorological, demographic, medical and health resource, and economic aspects and TB incidence are lacking. Hence, this study aims to bridge this gap by examining the nationwide perspective to better understand the relationship between macro-influence factors and TB incidence, facilitating more targeted interventions.

## Methods

### Data collection

The surveillance data for TB incidence in Chinese mainland from 2014 to 2021 were obtained from the NNDRS, an internet-based real-time disease-reporting system. The reported cases encompass suspected case, clinically diagnosed cases and etiologically confirmed cases, aligning with the diagnosis criteria for TB stipulated and disseminated by the National Health Commission of the People's Republic of China [15]. Suspected cases were excluded from the analysis, focusing on clinically diagnosed and etiologically confirmed cases. Anonymized data included demographic details (residential ID number, sex, age, and occupation) and clinical particulars (dates of symptom onset, diagnosis date, and diagnosis category).

Demographic data by age and sex for 31 provincial-level administrative divisions (PLADs) and the Chinese mainland were collected from the National Bureau of Statistics of China (http://www.stats.gov.cn/english/Statisticaldata/AnnualData, accessed on April 20, 2023). Daily meteorological monitoring data during 2017–2019, including daily average temperature (°C, Atemp), daily average relative humidity (%, ARH), daily average wind speed (m/s, AWS), daily sunshine duration (h, SD), and daily precipitation (mm, PRE), were collected from the China Meteorological Data Sharing Service System (http://data.cma.cn/, accessed on April 22, 2023). Yearly province-level economic [gross domestic product (GDP) per capita], demographic (population, population density, sex ratio, natural population growth rate and urbanization rate), and medical and health resource data (number of medical and health institutions, number of health technicians per 10,000 population, number of beds in medical and health institutions per 10,000 population, total health expenses) during 2014–2019 were collected from the National Bureau of Statistics of China (http://www.stats.gov.cn/english/Statisticaldata/AnnualData, accessed on March 20, 2023). The definition of each indicators were in the Supplement Table 1.

The administrative regions were categorized into province-level, prefecture-level, county-level and township level administrative regions. This study focused on the 31 PLADs in Chinese mainland, stratified into seven regions. The period from disease onset to diagnosis was calculated as the time of diseases onset minus the time of diagnosis by medical and health institutions, and classified into nine groups: 0–6 days, 1–2 weeks, 2–3 weeks, 3 weeks–1 month, 1–2 months, 2–6 months, 6 months–1 year, 1–2 years and more than 2 years.

### Joinpoint regression analysis

Temporal trends were analyzed using Joinpoint regression software (version 4.9.0.0, National Cancer Institute, Rockville, MD, USA). The default modeling method was the grid search method, and Monte Carlo permutation testing was the default model optimization strategy. The Bayesian information criterion (*BIC*) was employed as a metric for gauging good fit [16]. The average annual percent changes (AAPCs) with their 95% confidence interval (*CI*) were calculated for incidence rates during 2014–2021, which was subsequently computerized as a geometrically weighted average of the generated annual percent changes (APCs). The APC serves as an indicator of the average annual percentage alteration in incidence rates and is represented by the slope of the fitted line of each interval. The APC is used to evaluate the internal trend of each independent interval of a segmented function, or a global trend with a number of connected points of zero. An AAPC/APC > 0 (*P* < 0.05) denotes an increasing trend in incidence rates, whereas an AAPC/APC < 0 (*P* < 0.05) signifies a decreasing trend. Conversely, *P* > 0.05 indicates the trends stable. The APC can be expressed as Eq. (1), where *y* represents the incidence rate, *x* represents year,* β*_*1*_ represents regression coefficient.

$$\ln (y) = \beta_{0} + \beta_{1} x$$

1 $$APC = \left[ {\frac{{{\text{y}}_{x + 1} - y_{x} }}{{y_{x} }}} \right] \times 100 = (e^{{\beta_{1} }} - 1) \times 100$$

### Spatiotemporal analysis

Spatiotemporal analysis was conducted using SaTScan software (version 10.1, Kulldorff and Information Management Services, Inc., Boston, MA, USA). A Poisson probability model identified clusters of TB with a temporal and spatial window of 30% [17]. By juxtaposing observed and predicted events within each location window, assuming a random distribution, probable clusters were pinpointed. The cluster exhibiting the highest log-likelihood ratio (*LLR*) was deemed the most likely cluster, while others were ranked as secondary clusters in a specific sequence [17]. Relative risk (*RR*) indicated the elevated infection risk within the cluster compared to outside it [17]. Spatiotemporal analysis was performed at both province-level and prefecture-level during 2014–2021.

### Distributed lag non-linear model (DLNM)

A two-stage DLNM was used to analyzed relationship between meteorological factors and TB incidence based on the daily data during 2017–2019. The first stage involved constructing DLNM at each prefecture-level site to assess the lag and non-linearity of factors on TB risk. The DLNM based on a quasi-Poisson distribution served as the basic model for detecting possible delayed effects and nonlinear associations between exposures and TB incidence for each city in the first stage of analysis. The DLNM can be expressed as Eq. (2), where *Y*_*t*_ represents the outcome variable, which conforms to a normal distribution, Gamma distribution, or Poisson distribution; E (*Y*_*t*_) represents the expectation of the dependent variable *Y* at time *t*; *g* represents the connection function; *s*_*j*_ represents the nonlinear function between *x*_*j*_ and E (*Y*_*t*_); *u*_*k*_ represents other variables that have a linear relationship with E(*Y*_*t*_); *β, γ* represents the parameter vectors of *x*_*j*_ and *u*_*k*_ respectively [18, 19].

2 $${\text{g}}(u_{t} ) = \alpha + \sum\nolimits_{j = 1}^{j} {s_{j} (} x_{tj} ;\beta_{j} ) + \sum\nolimits_{k = 1}^{k} {\gamma_{k} u_{tk} }$$

In the second stage, a multivariate meta-regression model was constructed to capture the overall pooled exposure–response relationship in Chinese mainland [18, 20]. The cumulative effects of each independent variable on TB incidence were calculated, then lag-specific effects were calculated in different levels of variable. The crossbasis functions of Atemp, ARH, SD, PRE and AWS were built to analyze the lag-exposure–response relationship of meteorological factors. When one factor was included in the function, the other variables were set as covariates. The variance inflation factor (*VIF*) of meteorological factors were calculated to judge the multicollinearity between variables in different models.

The degree of freedom (*df*) of spline function of meteorological factors was set to three. Some studies have reported that the average incubation period of TB ranges from four to eight weeks [14], we set the maximum lag as 60 days. The sensitivity analysis was conducted by adjusting three aspects of parameters to test the robustness of our results, including the *df* of crossbasis (*df* = 3–5), the *df* of time variable (*df* = 6–8) and the lag days (lag = 55, 60, 65). The different quantiles of each independent variable (P_5_, P_25_, P_75_, and P_95_) were defined as extreme low, low, high, and extreme high levels. On the basis of the above model, taking the median of each factor as the reference value, the influence of meteorological factors on TB was discussed. *RR* is a measure of association which represents the change in TB incidence risk at any given Atemp compared with a reference Atemp (median value) [21], as well as for ARH, AWS, SD and PRE. The attributable fraction (*AF*) is a measure that quantifies the public health impact of an exposure on a factor. The *AF* of low and high values were calculated for each prefecture-level site and then the overall *AF* was estimated. Low value refers to value below the median (P_50_), dividing into mild low value (P_5_–P_50_) and extreme low value (< P_5_). High value refers to value above the median (P_50_), dividing into mild high value (P_50_–P_95_) and extreme high value (> P_95_).

The analysis mentioned above was conducted by R (version 4.0.3, R Development Core Team, USA), with package “dlnm” [22] and “splines” (R Core Team, 2021) to fit all DLNMs and the package “mvmeta” to conduct all multivariate meta-regression models.

### Spatial panel data model

A spatial panel yearly data model at each province-level site were constructed to evaluate the impacts of factors from demographic, medical and health resource, and economic aspects on TB incidence. A spatial autocorrelation analysis using Moran's *I* and scatter plot was performed to test if there is a spatial correlation between regions, followed by spatial panel estimations with suitable models. We adopted the data during 2014 to 2019 for spatial panel model analysis. All variables were taken as logarithmic values.

### Spatial autocorrelation analysis

Spatial dependence is a geographical phenomenon. The regional TB incidence has the characteristics of spatial spillover and spatial diffusion, with a great impact on the incidence of neighboring areas. The Moran's *I* was selected to measure the spatial autocorrelation between the incidence rate of TB in different regions. The value of Moran's *I* range is from -1 to 1, with Moran's *I* being < 0, = 0 and > 0 indicating the presence of spatial negative, no and positive autocorrelation, respectively. The Moran's *I* is defined as Eq. (3), where *N* is the number of spatial units indexed by locations (PLADs in this study) *i* and *j*, *W*_*ij*_ is a spatial weight matrix, *y*_*i*_ and *y*_*j*_ refer to the observations of *i* and *j*, respectively, *y* refers to the incidence rate of TB, $\overline y$ refers to the mean of *y*.

3 $$I = \frac{{\sum\nolimits_{{{\text{i}} = 1}}^{N} {\sum\nolimits_{j \ne 1}^{N} {W_{ij} } \left( {y_{i} - \overline{y}} \right)\left( {y_{j} - \overline{y}} \right)} }}{{S^{2} \left( {\sum\nolimits_{i = 1}^{N} {\sum\nolimits_{j = 1}^{N} {W_{ij} } } } \right)}} = \frac{{\sum\limits_{i = 1}^{N} {\sum\limits_{j \ne 1}^{N} {W_{ij} \left( {y_{i} - \overline{y}} \right)\left( {y_{j} - \overline{y}} \right)} } }}{{\left( {\sum\limits_{i = 1}^{N} {\sum\limits_{j \ne 1}^{N} {W_{ij} } } } \right)\sum\limits_{i = 1}^{N} {\left( {y_{i} - \overline{y}} \right)^{2} } }}$$

The spatial panel data model defines the correlation mode and degree between research units by introducing a spatial weight matrix. A spatial weight matrix is necessary for providing spatial-structure information between adjacent areas and how they interact with each other. Here, the Rook weight matrix was adopted. The spatial weight matrix is defined as *W*, with elements *W*_*ij*_ indicating whether or not observations *i* and *j* are spatially close. If units *i* and *j* (≠ *i*) are neighbors, the spatial weight is 1; otherwise, it is 0. *W*_*ij*_ can be written as Eq. (4).

4 $$W_{ij}=\left\{\begin{array}{l}1\\0\end{array}\right.if\;i\;is\;contiguous\;to\;j,\;W_{ij}=1;\;otherwise\;W_{ij}=0$$

GeoDa (version 1.18.0, Luc Anselin, Urbana) was used for calculating the Moran's *I* and drawing the Moran scatter plots, as well as constructing Rook spatial weight matrix based on the contiguity for 31 PLADs in Chinese mainland.

### Spatial panel models

The spatial panel models can effectively solve the spatial dependence of TB incidence rate. Three types of spatial panel models were considered, including the spatial lag model (SLM), the spatial error model (SEM) and spatial Durbin model (SDM). The SLM can be interpreted the spatial dependency between the dependent variables, and can be written as Eq. (5) [23]. The δ is the spatial autoregressive coefficient and *W*_*ij*_*′* is the row standardized spatial weight matrix (*W*_*ij*_).

5 $$y_{it} = \delta \sum\limits_{j = 1}^{N} {W_{ij} } ^{\prime}y_{jt} + \beta x_{it} + u_{i} + \varepsilon_{it}$$

The SEM considers spatial lag error term, and can be written as Eq. (6) [23]. The *λ* refers to the spatial autocorrelation coefficient. *ϕ*_*it*_ reflects the spatially autocorrelated error term.

6 $$\begin{gathered} y_{it} = \beta x_{it} + u_{i} + \phi_{it} \hfill \\ \phi_{it} = \lambda \sum\limits_{j = 1}^{N} {W_{ij} ^{\prime}\phi_{it} + \varepsilon_{it} } \hfill \\ \end{gathered}$$

The SDM can be used to investigate not only the influence of local variables on dependent variables but also the influence of adjacent regional dependent variables and their independent variables, and can be expressed as Eq. (7) [24].

7 $$y_{it} = \delta \sum\limits_{{{\text{j}} = 1}}^{N} {W_{ij} ^{'}\,y_{it} + } \beta x_{it} + \gamma \sum\limits_{j = 1}^{N} {W_{ij} ^{'}\,x_{jt} + } u_{i} + \varepsilon_{it}$$

### Model selection

The SDM was selected as the base model, and conducted LR and Wald test to determine whether SDM can degenerate into SLM or SEM. If *P* < 0.05, the SDM was selected; otherwise SLM or SEM were selected. The lagrange multiplier test (LM test) was applied for testing if there is a spatial error effect and a spatial lag effect, including four tests (LM-lag, LM-error, robust LM-lag, and robust LM-error tests). The selection of fixed-effect and random-effect models was determined by the objective of this study and Hausman test. We focused on the analysis in 31 PLADs and did not extrapolate the results, so we chose the fixed effects model [13]. The Akaike information criterion (*AIC*), *BIC*, and *R*^*2*^ were compared between time fixed, individual fixed and two-way fixed SDM to select suitable model. The Stata (version 17.0, Stata Corporation, College Station, Texas) was used for panel spatial regression analyses.

## Results

### Overview of TB cases

**Fig. 1:**
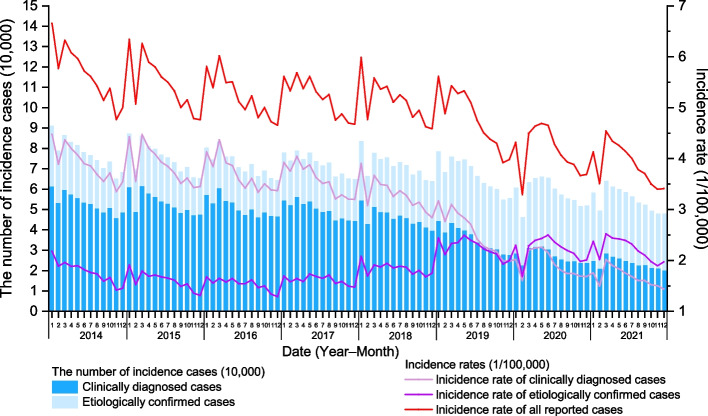
The monthly TB incidence cases and rates in Chinese mainland during 2014–2021

A total of 6,587,439 TB cases were reported in Chinese mainland from 2014 to 2021, with an average annual incidence rate of 59.17/100,000. Among them, 4,073,251 cases were clinically diagnosed cases (average annual incidence rate: 36.59/100,000). While 2,514,188 cases were etiologically confirmed cases (average annual incidence rate: 22.58/100,000), accounting for 38.17% of all reported TB cases (Fig. 1).

### Demographic distributions of TB cases

**Fig. 2:**
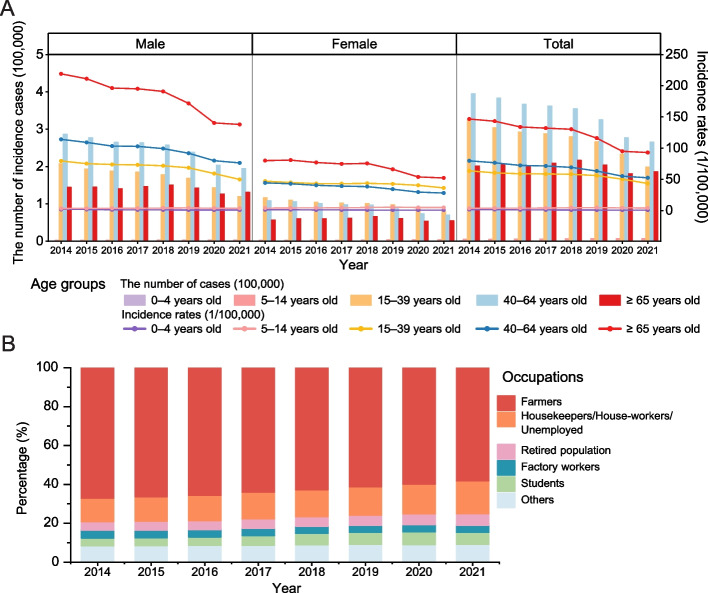
The demographic distributions of TB cases in Chinese mainland during 2014–2021. **A** The TB incidence number and rates by gender and age groups. **B** The percentage of TB cases by occupations

There were 4,535,201 male cases and 2,052,238 female cases during 2014–2021, with average annual incidence rates of 79.60/100,000 and 37.75/100,000, respectively (Fig. 2A). Both male (AAPC = -5.26%, 95% *CI*: -6.21, -4.45%) and female (AAPC = -4.78%, 95% *CI*: -5.78, -3.90%) incidence rates showed a decreasing trend over the years (Additional file: Supplement Table 2). Age distribution showed that the highest number of TB cases was in the 40–64 age group, followed by 15–39 age group; the highest incidence rate was in population aged 65 years and older (annual average incidence rate was 121.36/100,000), followed by 40–64 (67.53/100,000) and 15–39 (56.44/100,000) age groups (Fig. 2A). Incidence rates generally decreased from 2014 to 2021 across different age groups, except for an increase in the 5–14 age group (Additional file: Supplement Table 2). Incidence rates varied by age and gender, with a slightly higher incidence rate for females in the 15–39 age group and a higher incidence rate for males in the 40–64 age group (Fig. 2A).

Farmers accounted for 63.37% of all reported cases, followed by housekeepers/house-workers/unemployed (13.83%), students (5.20%), retired population (5.00%), and factory workers (3.79%). (Fig. 2B). Among all incidence cases, the proportion of farmers (AAPC = -1.77%, 95% *CI*: -1.90, -1.67%) and factory workers (AAPC = -1.44%, 95% *CI*: -2.02, -0.98%) decreased from 2014 to 2021, while the proportion of housekeepers/house-workers/unemployed (AAPC = 4.98%, 95% *CI*: 4.35, 5.48%), retired population (AAPC = 4.62%, 95% *CI*: 3.87, 5.30%) and students (AAPC = 7.94%, 95% *CI*: 5.20, 11.58%) increased (Additional file: Supplement Table 3).

### Temporal trends of TB cases

**Fig. 3:**
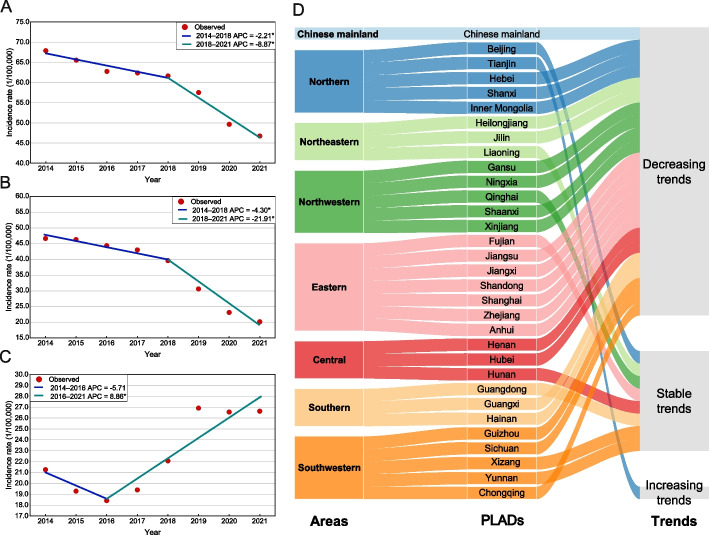
The temporal trends of incidence rates for 31 PLADs and Chinese mainland during 2014–2021. **A** The temporal trend of TB incidence rate in Chinese mainland. **B** The temporal trend of incidence rate for clinically diagnosed TB cases in Chinese mainland. **C** The temporal trend of incidence rate for etiologically confirmed TB cases in Chinese mainland. **D** The temporal trends of TB incidence rates in the 31 PLADs. Notes: In Figure **A**-**C**, points represent the observed incidence rates, lines represent the fitting line of the observed incidence rates and the slopes indicate the value of APC, * represents the *P* < 0.05. PLADs, Provincial-level administrative divisions; APC, Annual percent changes

The overall TB incidence rate of TB decreased from 67.05/100,000 in 2014 to 46.40/100,000 in 2021 (AAPC = -5.12%, 95% *CI*: -5.93, -4.41%), with obvious decrease from 2018 to 2021 (APC = -8.87%, 95% *CI*: -11.97, -6.85%) (Fig. 3A). The rate of clinically diagnosed TB cases was also with decreasing trend (AAPC = -12.29%, 95% *CI*: -14.56, -10.70%) (Fig. 3B). The incidence of etiologically confirmed cases decreased from 2014 to 2016 without statistically significance (APC = -5.71%, 95% *CI*: -13.65, 3.72%), then statistically increased from 2016 to 2021 (APC = 8.86%, 95% *CI*: 6.19, 16.32%) (Fig. 3C).

The temporal trends of TB incidence rates for 31 PLADs showed that 22 PLADs decreased with statistically significance, with the biggest decrease in Gansu (AAPC = -11.55%, 95% *CI*: -15.39, -8.19%). An increasing trend was observed in one province. In addition, a joinpoint was observed in eight PLADs with an irregular change during 2014–2021, of which four PLADs present a decreasing trend during the later period (Fig. 3D and Additional file: Fig. S1).

### Spatiotemporal distributions of TB cases

**Fig. 4:**
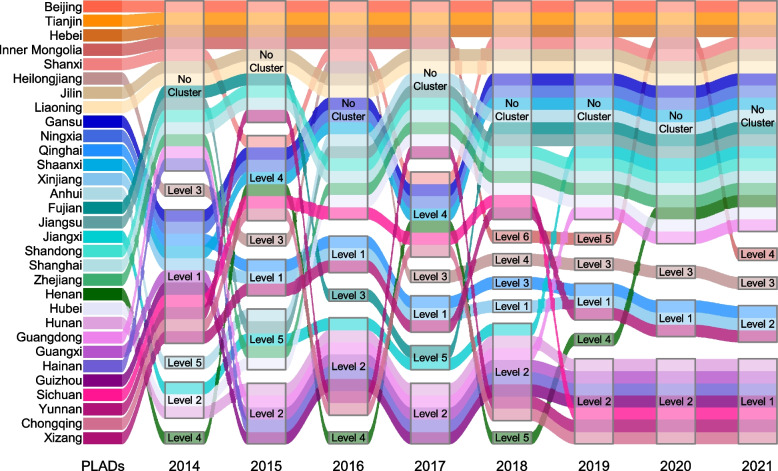
The spatiotemporal distributions of PLADs’ incidence rates in single years during 2014–2021. PLADs, Provincial-level administrative divisions

The spatiotemporal analysis during 2014–2021 based on province-level incidence rates identified six clusters covered 22 PLADs in different periods, with the most likely cluster located in Xinjiang, Qinghai and Xizang during March 2017–June 2019 (*RR*: 3.94, *P* < 0.001). Other five clusters distributed in different PLADs in different period (Table 1). The spatiotemporal analysis for single years showed that the level of clusters for most PLADs changed during 2014–2021, while remained unchanged for some PLADs (Fig. 4).

**Table 1:** The spatiotemporal distributions of PLADs' incidence rates during 2014–2021

**The level of clusters**	**Covered PLADs**	**The period of clusters**	***RR***	***LLR***	***P***
1	Xinjiang, Qinghai, Xizang	March 2017–June 2019	3.94	112946.67	< 0.001
2	Guangdong, Guizhou, Guangxi, Hainan, Hunan	January 2014–April 2016	1.61	62121.91	< 0.001
3	Heilongjiang	January 2014–April 2016	1.55	6669.02	< 0.001
4	Gansu, Henan, Ningxia, Shanxi, Shaanxi, Sichuan, Chongqing	January 2014–June 2015	1.14	2372.64	< 0.001
5	Anhui, Hubei, Jiangsu, Jiangxi, Zhejiang	January 2014–June 2014	1.12	599.34	< 0.001
6	Inner Mongolia	January 2018–May 2018	1.10	29.06	< 0.001

*PLADs* Provincial-level administrative divisions, *RR *Relative risk, *LLR* Log-likelihood ratio

The spatiotemporal analysis during 2014–2021 based on prefecture-level incidence rates identified 17 clusters covered 102 prefecture-level administrative divisions in different periods, with the most likely cluster located mainly in five prefecture-level administrative divisions (Kizilsu Kirghiz Autonomous Prefecture, Kashi Prefecture, Aksu Prefecture, Hotan Prefecture, and Tumxuk) of Xinjiang during March 2017–June 2019 (*RR* = 8.55, *P* < 0.01). Other significant clusters were mainly present in various eastern regions of China (Additional file: Supplement Table 4).

### The period from TB onset to diagnosis

**Fig. 5:**
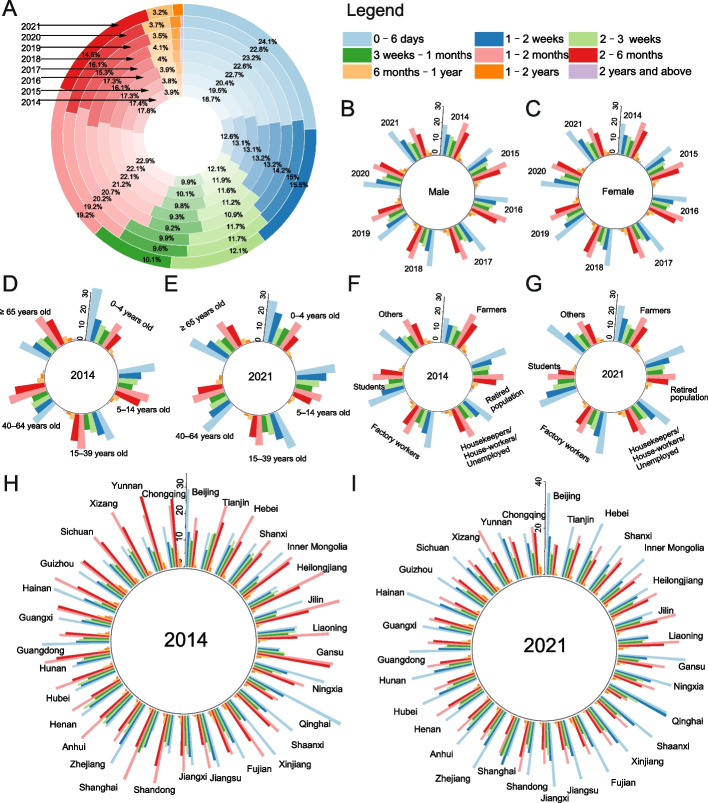
The period from TB onset to diagnosis by year, gender, age groups, occupations and PLADs. **A** The percentage of nine period groups in Chinese mainland during 2014–2021. **B** The percentage of nine period groups in male during 2014–2021. **C** The percentage of nine period groups in female during 2014–2021. D) The percentage of nine period groups by age groups in 2014. **E** The percentage of nine period groups by age groups in 2021. **F** The percentage of nine period groups by occupations in 2014. **G** The percentage of nine period groups by occupations in 2021. **H** The percentage of nine period groups by PLADs in 2014. **I** The percentage of nine period groups by PLADs in 2021. Notes: PLADs, provincial-level administrative divisions

The median period from TB onset to diagnosis was 23 [inter-quartile range (IQR): 8–51] days during 2014–2021, decreasing from 26 (IQR: 10–56) days in 2014 to 19 (IQR: 7–44) days in 2021. The dominant period shifted from 1–2 months in 2014–2016 to 0–6 days in 2017–2021. The proportion of 0–6 days increased from 18.70% in 2014 to 24.08% in 2021, as well as the proportion of 1–2 weeks increased from 12.56% in 2014 to 15.45% 2021 (Fig. 5A).

The 0–6 days period became dominant across genders, ages, and occupations. Among farmers, the dominant period changed from 1–2 months in 2014 to 0–6 days in 2021 (Fig. 5B-G). Three patterns were observed in the spatial distribution of the period in 2021, with the 0–6 days period dominant in 23 PLADs, the 1–2 months period dominant in six PLADs, and the 1–2 weeks period dominant in two PLADs. Changes in the dominant period were observed in 17 PLADs from 2014 to 2021 (Fig. 5H-I).

### The proportion of etiologically confirmed cases

**Fig. 6:**
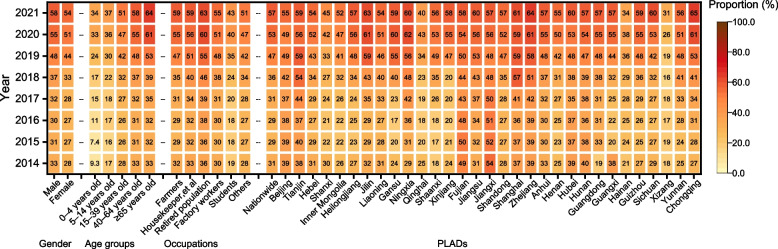
The proportion of etiologically confirmed cases by gender, age groups, occupations and PLADs during 2014–2021. Notes: PLADs, Provincial-level administrative divisions

The average proportion of etiologically confirmed cases during 2014–2021 was 38.17%. The proportion of etiologically confirmed cases increased during 2014–2021 (AAPC = 9.62%, 95% *CI*: 6.43, 14.61%), including changed irregularly initially (APC = -5.10%, 95% *CI*: -15.55, 12.42%) and then increased from 29.33% in 2016 to 56.98% in 2021 (APC = 16.13%, 95% *CI*: 9.66, 33.95%) (Fig. 6 and Additional file: Supplement Table 5). The proportion of etiologically confirmed cases among males was consistently higher than among females in each year. Both genders showed an increase in the proportion of etiologically confirmed cases, with males rising from 32.62% in 2014 to 58.23% in 2021 (AAPC = 9.30%, 95% *CI*: 6.32, 13.98%), and females rising from 28.38% in 2014 to 54.28% in 2021 (AAPC = 11.40%, 95% *CI*: 8.14, 15.25%). The proportion of etiologically confirmed cases increased with age, with the highest proportion in the population aged 65 years and older. Proportions generally increased in all age groups, especially in the 0–4 age group (AAPC = 25.50%, 95% *CI*: 18.97, 32.48%). The retired population had the highest proportion of etiologically confirmed cases among occupational categories, while students had the lowest. The proportion of etiologically confirmed cases increased among six occupation categories, particularly in students (APC = 13.34%, 95% *CI*: 9.64, 19.27%) (Fig. 6 and Additional file: Supplement Table 5).

In 2021, the proportion of etiologically confirmed cases in 15 PLADs was higher than the nationwide average (56.98%). The proportion increased for 31 PLADs, especially in Shaanxi from 2014 to 2021 (AAPC = 22.66%) (Fig. 6 and Additional file: Supplement Table 5).

### Relationships between meteorological factors and TB incidence

**Fig. 7:**
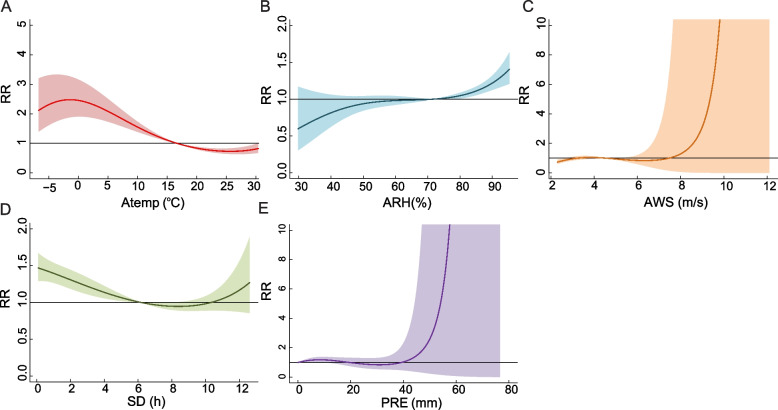
The cumulative effects of Atemp, ARH, AWS, SD and PRE on the risk of TB incidence. Notes: *RR*, Relative risk; Atemp, Average temperature; ARH, Average relative humidity; AWS, Average wind speed; SD, Sunshine duration; PRE, Precipitation. The median value was reference

There was no multicollinearity between five meteorological factors (Atemp, ARH, AWS, SD and PRE) based on the variance inflation factor (*VIF*) (Additional file: Supplement Table 6). Overall pooled cumulative exposure–response relationships of exposure to meteorological indicators with TB incidence were identified (Fig. 7). A risk effect was observed when Atemp lower than median value (16.69℃) and the protective effect when Atemp higher than 16.69℃, with the *RR* peaked at -1.5℃ (*RR*: 2.48, 95% *CI*: 1.88, 3.27) (Fig. 7A). A risk effect was observed when ARH higher than median value (71.73%), with the *RR* peaked at 95% (*RR*: 1.33, 95% *CI*: 1.18, 1.49) (Fig. 7B). A protective effect was observed when AWS lower than 2.79 m/s (Fig. 7C). A risk effect was observed when SD lower than median value (6.18 h), with *RR* peaked at 0 h (*RR*: 1.52, 95% *CI*: 1.33, 1.74) (Fig. 7D). A risk effect was observed when PRE lower than 13.2 mm, with *RR* peaked at 9 mm (*RR*: 1.26, 95% *CI*: 1.08, 1.48) (Fig. 7E). The sensitivity analyses showed the similar results with the varying degrees of freedom of cross basis and time variable, lag days (Additional file: Fig. S2).

**Fig. 8:**
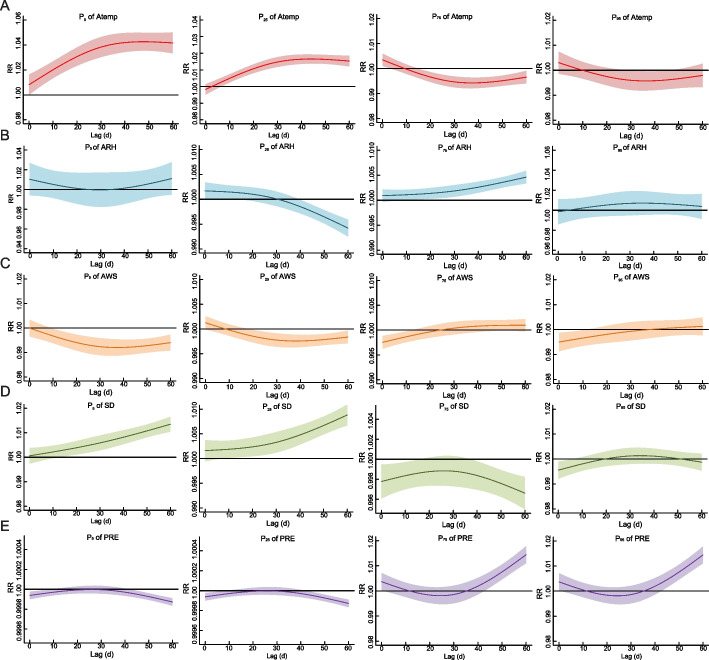
The lag-response curves for P_5_, P_25_, P_75_, P_95_ of variables on TB incidence at lag 1–60 days. Notes: P_5_, the 5th percentile; P_25_, the 25th percentile; P_75_, the 75th percentile; P_95_, the 95th percentile; Atemp, Average temperature; ARH, Average relative humidity; AWS, Average wind speed; SD, Sunshine duration; PRE, Precipitation; *RR*, Relative risk. The median value was reference

Extreme low Atemp (< -6.76℃) had a risk effect on TB at lag 1 days and later, and *RR* peaked at lag 42 days (*RR*: 1.04, 95% *CI*: 1.04, 1.05). Low Atemp (< 7.43℃) had a risk effect on TB at lag 6 days and later. High Atemp (> 23.65℃) had a risk effect at lag 1–4 days, and a protective effect at lag 15–60 days. Extreme high Atemp (> 29.06℃) had a protective effect at lag 30–46 days (Fig. 8A). Low ARH (< 56.52%) had a risk effect at lag 1–16 days, and had a protective effect at lag 42 days and later. High ARH (> 82.17%) had a risk effect on TB at lag 14 days and later (Fig. 8B). The protective effect of extreme low (< 2.79 m/s), low (< 3.65 m/s), high (> 5.71 m/s), and extreme high (> 8.24 m/s) AWS on TB at lag 12 days later, 20 days later, 1–12 days and 1–12 days respectively (Fig. 8C). Extreme low (< 0.004 h) and low SD (< 6.18 h) had a risk effect at lag 10 days and later. High SD (> 8.75 h) had a protective effect at 1–16 days and 39–60 days, and extreme high SD (> 11.19 h) had a protective effect at 1–6 days (Fig. 8D). High (> 1.78 mm) and extreme high (> 14.74 mm) PRE had a risk effect at 44–60 days and 42–60 days respectively (Fig. 8E).

**Fig. 9:**
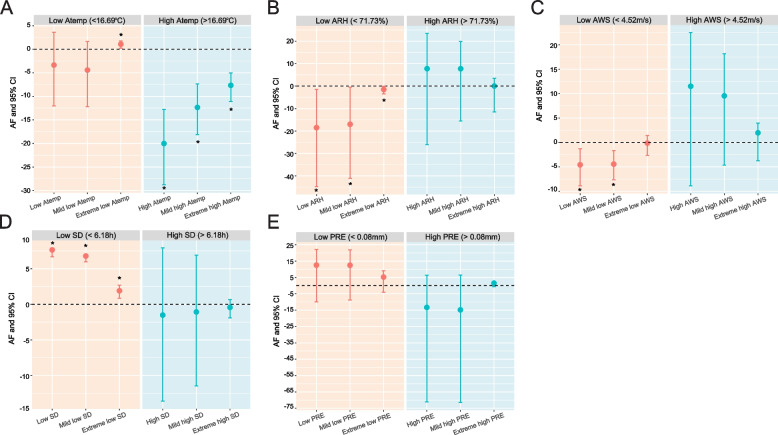
The *AF* of Atemp, ARH, AWS, SD and PRE on the risk of TB incidence. Notes: Low value refers to value below the median (P_50_), dividing into mild low value (P_5_–P_50_) and extreme low value (< P_5_). High value refers to value above the median (P_50_), dividing into mild high value (P_50_–P_95_) and extreme high value (> P_95_). *AF*, Attributable fraction; Atemp, Average temperature; ARH, Average relative humidity; AWS, Aaverage wind speed; SD, Sunshine duration; PRE, Precipitation; P_5_, the 5th percentile; P_50_, the 50th percentile; P_95_, the 95th percentile; *CI*, Confidence interval. The median value was reference

Extreme low Atemp (< -6.76 ℃) contributed to 1.07% of all TB incidence, and high Atemp (> 16.69 ℃) was associated with a 20.02% decrease in TB incidence (Fig. 9A). Low ARH (< 71.73%) attributable to 18.50% of the TB incidence decrease, while high ARH(> 71.73%) attributable to 7.76% of TB incidence without statistical significance (Fig. 9B). The low AWS (< 4.52 m/s) attributable to 4.59% of the TB incidence decrease, while the high AWS (> 4.52 m/s) attributable to 11.50% of the TB incidence without statistical significance (Fig. 9C). The low SD (< 6.18 h) attributable to 7.64% of the TB incidence (Fig. 9D). The low PRE (< 0.08 mm) attributable to 12.51% of the TB incidence and high PRE (> 0.08 mm) attributable to 13.33% of the TB incidence decrease, both without statistical significance (Fig. 9E).

### Relationships between demographic, medical and health resources, and economic factors and TB incidence

The Moran's *I* statistics and Moran scatter plots showed the positive autocorrelation of TB incidence rates, indicating that a spatial panel data model should be used (Additional file: Supplement Table 7 and Fig. S3). The SDM with a time fixed effect was chosen based on LM, LR and Wald tests, *AIC*, *BIC* and *R*^*2*^ of models (Additional file: Supplement Tables 8–10).

The SDM with a time fixed effect showed positive associations between TB incidence rates and sex ratio (*β* = 1.98), number of beds in medical and health institutions per 10,000 population (*β* = 0.90), and total health expenses (*β* = 0.55). There were negative associations between TB incidence rates and population (*β* = -1.14), population density (*β* = -0.19), urbanization rate (*β* = -0.62), number of medical and health institutions (*β* = -0.23), and number of health technicians per 10,000 population (*β* = -0.70). No statistically significant correlation was found between TB incidence rates and GDP, natural population growth rate (*P* > 0.05) (Table 2).

**Table 2:** The effects of demographic, medical and health resource, and economic factors on TB incidence by SDM with a time fixed effect

**Variables**	***β***	***95% CI***	***Z***	***P***
Log_10_(population)	-1.14	-1.94, -0.34	-2.78	0.005
Log_10_(population density)	-0.19	-0.28, -0.10	-4.31	< 0.001
Log_10_(natural population growth rate)	-0.01	-0.04, 0.02	-0.75	0.46
Log_10_(urbanization rate)	-0.62	-1.21, -0.03	-2.07	0.04
Log_10_(sex ratio)	1.98	0.71, 3.25	3.05	0.002
Log_10_(number of medical and health institutions)	-0.23	-0.41, -0.06	-2.67	0.008
Log_10_(number of health technicians per 10,000 population)	-0.70	-1.30, -0.09	-2.27	0.02
Log_10_(number of beds in medical and health institutions per 10,000 population)	0.90	0.35, 1.45	3.19	0.001
Log_10_(total health expenses)	0.55	0.06, 1.05	2.18	0.03
Log_10_(GDP)	-0.20	-0.49, 0.09	-1.35	0.18
rho	0.27	0.07, 0.47	2.63	0.008
sigma2_e	0.05	0.04, 0.07	9.56	< 0.001

*SDM* Spatial Durbin model, *CI* Confidence interval

## Discussion

This groundbreaking nationwide study assesses the relationship between TB incidence and various factors, encompassing meteorological, demographic, medical and health resource, and economic aspects, utilizing China's national surveillance data. It also delves into TB epidemiological characteristics and diagnostic capabilities. *Key findings are as follows:*

Noteworthy declines were observed in TB incidence rates from 2014 to 2021, the diagnostic ability have improved significantly. The great achievement is attributable to mass efforts and public health interventions, for instance, TB prevention and control strategy, the increasing TB funds, the improvement of surveillance, and social progress [25–27]. However, there were still some higher risks among males, farmers and individuals aged 65 years and older, as well as some geographical locations. Complex interactions among biological, social, cultural, and economic factors contribute to gender and age variations in TB incidence [28, 29]. The farmers account for the majority of all reported cases, which consistent with other studies [8, 30]. The physical condition, limited health services and patient management in rural areas, and a shortage of TB awareness could contribute to the higher TB incidence among the farmer [31].

Spatiotemporal analysis showed that the clusters with higher incidence were mainly in several PLADs in western areas. The limited health services and patient management, less developed socioeconomic infrastructure, and inconvenient transportation conditions in western areas increase the difficulties for TB control and care [8]. Importantly, the TB incidence of most PLADs showed a downward trend, of which Gansu, Guangxi, and Xinjiang were with higher AAPC. The faster decline in these areas is more likely attributed to comprehensive interventions [25, 32]. For example, the incidence of Gansu, which is one of the PLADs with lower socioeconomic conditions, decreased faster. The main reason is that the government has taken a series of intervention measures to control TB, such as strengthening the monitoring and reporting system, improving the diagnosis and treatment of TB, and increasing funding investment [32]. With economic development and social progress, people's living standards have improved, all of which contribute to reducing the incidence rate of TB. Continuous efforts are still needed to end TB as soon as possible.

Improvements in diagnostic ability and reporting were evident, with a shortened period from TB onset to diagnosis, especially in farmers. The early diagnosis was greatly benefit for promoting the treatment success and reducing TB transmission. The period of most PLADs shortened from 2014 to 2021, but remained unchanged in several PLADs which should be further strengthened the efforts. Furthermore, the proportion of etiologically confirmed cases increased from 31.31% in 2014 to 56.98% in 2021. The males and the older were with the higher proportion of etiologically confirmed cases, which is great benefit for TB control among the high-risk populations. It is worth noting that the proportion of etiologically confirmed cases remains low in some western regions with high incidence rates. So, it’s urgent to develop the simple, accurate and suitable TB diagnostic tools for earlier TB detection and intervention effectively.

Although only limited evidence is currently available regarding the association between meteorological factors and TB incidence at the nationwide in China, the findings of the present study are basically consistent with existing reports that Atemp, ARH, AWS, PRE and SD were associated with TB incidence [4, 10, 11, 14, 33, 34]. A study conducted in 16 cities in Anhui province reported that low temperature increased the risk of TB hospitalizations [34]. Our nationwide study, basing on prefecture-level and daily time scales, found low Atemp (< 16.69 ℃), high ARH (> 71.73%), and low SD (< 6.18 h) increased the risk of TB incidence. Previous study showed temperature and relative humidity were found as the vital factors for the formation of droplet diameter influencing the containing pathogens [35]. Clearly, *Mycobacterium tuberculosis* (*Mtb*), the pathogen of TB, is more likely to survive in the environment with low temperature and high humidity [10]. The short sunshine duration could reduce the UV rays, increasing the degree of low temperature and high relatively humidity, which contribute to *Mtb* survive in the environment. Also the specific meteorological conditions may influence the human body function and immunity [34, 36]. We found extreme low wind speed decrease the risk of TB incidence. But a previous study reported the low wind speed is a risk factor of TB, the high wind speed is a protective factor of TB [10]. The wind speed is a biphasic factor for TB incidence, which is difficult to clarify the impact on TB incidence. On the one hand, wind speed was benefit for the spreading of *Mtb*; on the other hand, wind speed could facilitate air circulation to avoid infection [37].

Several studies shown that urbanization [37], the number of health physicians [13], population density [30], and number of beds in medical institution [13, 30] are associated with the TB incidence. We explored the ten factors from demographic, medical and health resource, and economic aspects. Population, population density and urbanization rate were negatively related to TB incidence, which is consistent with the previous study [30]. The PLADs with better development level and economic situations are usually with higher population density and urbanization rate, leading to the higher accessibility for medical services and better living standards [13, 30]. The number of medical and health institutions, number of health technicians per 10,000 population were negatively related to TB incidence, indicating higher accessibility for medical services contribute to the TB control. The total health expenses is a factor reflects health and economic aspects. We found total health expenses was positively related to TB incidence, indicating the areas with higher TB incidence were prone to higher total health expenses. The total health expenses reflects the importance and cost burden level of health care by the government, society, and individual residents under certain economic conditions, as well as the main characteristics of health financing models and the fairness and rationality of health financing [38]. So, the improved investment and capacity-building for medical and health construction would benefit for TB control and prevention. The data was from passive surveillance system, the potential variations in reporting across regions and levels are limitations in this study.

## Conclusions

China has made great achievements in TB control and prevention, but challenges persist in specific populations and regions. This study emphasizes the importance of addressing meteorological, demographic, medical and health resource, and economic factors on TB incidence. Based on the findings, combining with the situation in different areas, the comprehensive digital/intelligent surveillance and response should be strengthened for earlier detecting the risk factors and taking interventions effectively.

### Supplementary Information

Additional file 1: Fig. S1. The temporal trends of 31 PLADs’ incidence rates during 2014–2021. Fig. S2. The sensitivity analysis results of DLNM for five meteorological factors. Fig. S3. The Moran’s I scatter plots in 2019.

Additional file 2: Supplement Table 1. The information of indicators from demographic, medical and health resource, and economic aspects. Supplement Table 2. The temporal trends of TB incidence rates among subpopulations from 2014 to 2021. Supplement Table 3. The temporal trends of proportion among different occupation from 2014 to 2021. Supplement Table 4. The spatiotemporal analysis for TB prefecture-level incidence rates in Chinese mainland. Supplement Table 5. The temporal trends of the proportion for etiologically confirmed cases from 2014 to 2021. Supplement Table 6. The VIF of five meteorological factors in the model. Supplement Table 7. The Moran's I statistics for TB incidence rates from 2014 to 2019. Supplement Table 8. The results of LM test. Supplement Table 9. The results of model selection. Supplement Table 10. The selection of three SDM.

## Data Availability

The data is from the National Notifiable Disease Reporting System (NNDRS), and the application is based on the requirement of NNDRS.

## Abbreviations

TB: Tuberculosis
NNDRS: National Notifiable Disease Reporting System
DLNM: Distributed Lag Nonlinear Model
PLADs: Provincial-level Administrative Divisions
Atemp: Average Temperature
ARH: Average Relative Humidity
AWS: Average Wind Speed
SD: Sunshine Duration
PRE: Precipitation
GDP: Gross Domestic Product
*BIC*: Bayesian Information Criterion
AAPCs: Average Annual Percent Changes
*CI*: Confidence Interval
APCs: Annual Percent Changes
*LLR*: Log-likelihood Ratio
*RR*: Relative Risk
*VIF*: Variance Inflation Factor
*df*: Degree of Freedom
SLM: Spatial Lag Model
SEM: Spatial Error Model
SDM: Spatial Durbin Model
LM test: Lagrange Multiplier test
IQR: Interquartile Range
*AIC*: Akaike Information Criterion
*Mtb*: *Mycobacterium tuberculosis*
