# Supplementary material for: Epidemiological characteristics of tuberculosis incidence and its macro-influence factors in Chinese mainland during 2014–2021
Source: Infect Dis Poverty. 2024 May 21;13:34. doi: 10.1186/s40249-024-01203-6 (PMC11107005; doi:10.1186/s40249-024-01203-6)
Supplement: Supplementary file 1 — Additional file 1: Fig. S1. The temporal trends of 31 PLADs’ incidence rates during 2014–2021. Fig. S2. The sensitivity analysis results of DLNM for five meteorological factors. Fig. S3. The Moran’s I scatter plots in 2019. [file 40249_2024_1203_MOESM1_ESM.pdf]

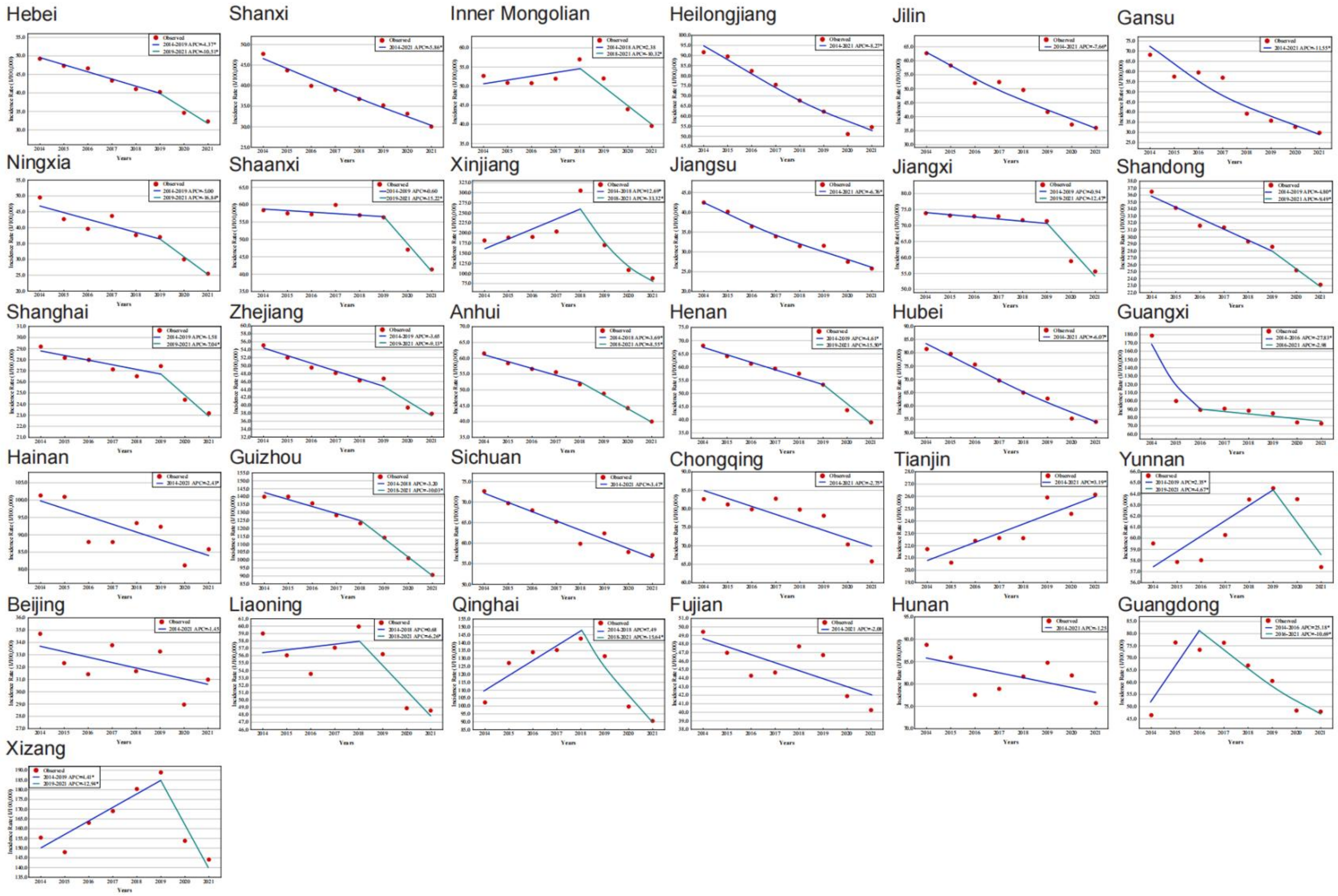

Additional file: Fig. S1. The temporal trends of 31 PLADs' incidence rates during 2014–2021. Notes: PLADs, provincial-level administrative divisions; APC, annual percent changes.

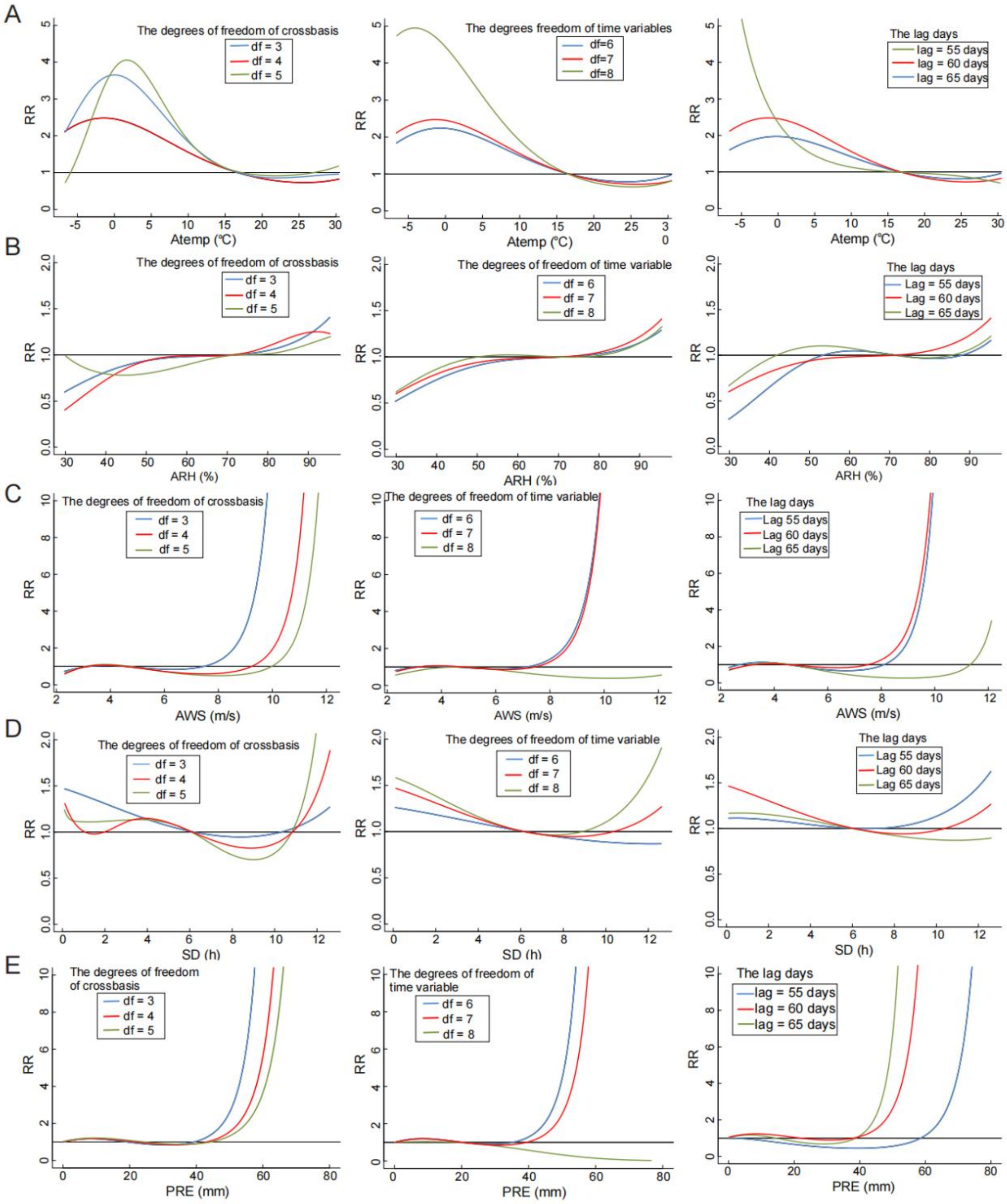

Additional file: Fig. S2. The sensitivity analysis results of DLNMs for five meteorological factors. Notes: DLNM, distributed lag nonlinear model; *RR*, relative risk; *df*, degrees of freedom.

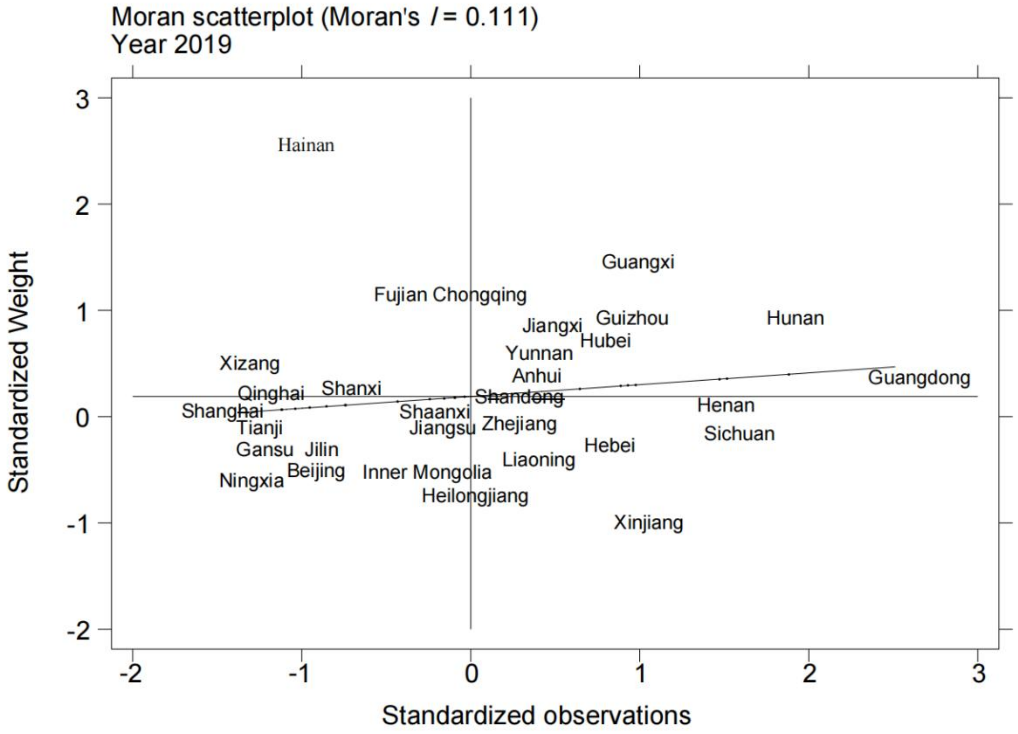

Additional file: Fig. S3. The Moran's *I* scatter plots in 2019.
