# Supplementary material for: Epidemiological characteristics of tuberculosis incidence and its macro-influence factors in Chinese mainland during 2014–2021
Source: Infect Dis Poverty. 2024 May 21;13:34. doi: 10.1186/s40249-024-01203-6 (PMC11107005; doi:10.1186/s40249-024-01203-6)
Supplement: Supplementary file 2 — Additional file 2: Supplement Table 1. The information of indicators from demographic, medical and health resource, and economic aspects. Supplement Table 2. The temporal trends of TB incidence rates among subpopulations from 2014 to 2021. Supplement Table 3. The temporal trends of proportion among different occupation from 2014 to 2021. Supplement Table 4. The spatiotemporal analysis for TB prefecture-level incidence rates in Chinese mainland. Supplement Table 5. The temporal trends of the proportion for etiologically confirmed cases from 2014 to 2021. Supplement Table 6. The VIF of five meteorological factors in the model. Supplement Table 7. The Moran's I statistics for TB incidence rates from 2014 to 2019. Supplement Table 8. The results of LM test. Supplement Table 9. The results of model selection. Supplement Table 10. The selection of three SDM. [file 40249_2024_1203_MOESM2_ESM.pdf]

Supplement Table 1. The information of indicators from demographic, medical and health resource, economic aspects

Aspects	Indicators	Defination
Demographic factors	Population	refers to the total number of individuals at a certain time and within a certain area
	Population density	refers to the number of people per unit of land area during a certain period of time. Calculation formula: Population density=population of a certain area/land area of that area (person/square kilometer)
	Sex ratio	refers to the ratio of the number of males to females. Calculation formula: Sex ratio=number of males/number of females *100
	Natural population growth rate	refers to the birth rate minus the mortality rate
	Urbanization rate	refers to the proportion of urban residents in the total resident population
Medical and health resource factors	Number of medical and health institutions	refers to the total number of hospitals, grassroots medical and health institutions, professional public health institutions, and other medical and health institutions
	Number of health technicians per 10,000 population	refers to the number of health professionals per 10000 population
	Number of beds in medical and health institutions per 10,000 population	refers to the total number of beds in medical and health institutions per 10000 people
	Total health expenses	refers to the monetary total amount of health resources raised by a country or region from the whole society during a certain period of time to carry out health service activities
Economic factor	Gross domestic product (GDP) per capita	refers to the GDP of a country or region divided by its population

Supplement Table 2. The temporal trends of incidence rates among subpopulations from 2014 to 2021

Variables	Whole		Period 1			Period 2		
	AAPC	95%*CI*	Time frame	APC1	95%*CI*	Time frame	APC2	95%*CI*
Male	-5.26*	-6.21, -4.45	2014–2018	-2.23	-3.73, 0.11	2018–2021	-9.16*	-12.74, -6.96
Female	-4.78*	-5.78, -3.90	2014–2018	-2.11	-3.50, 0.45	2018–2021	-8.22*	-12.20, -6.15
0–4 years old	-9.88*	-12.49, -7.55	—	—	—	—	—	—
5–14 years old	2.01*	1.11, 3.05	2014–2019	5.48*	4.35, 7.41	2019–2021	-6.18*	-9.50, -1.74
15–39 years old	-4.98*	-6.22, -4.03	2014–2019	-2.09*	-3.07, -0.18	2019–2021	-11.85*	-16.37, -7.50
40–64 years old	-5.91*	-6.55, -5.31	2014–2018	-3.48*	-4.64, -1.90	2018–2021	-9.06*	-11.64, -7.39
≥65 years old	-6.72*	-7.92, -5.55	2014–2018	-3.24	-5.19, 0.39	2018–2021	-11.18*	-15.69, -8.39

Notes: APC, annual percent changes; AAPC, average annual percent changes; *CI*, confidence interval. * represents the $P < 0.05$

Supplement Table 3. The temporal trends of incidence proportion among different occupation from 2014 to 2021

Variables	Whole		Period 1			Period 2		
	AAPC	95%*CI*	Time frame	APC1	95%*CI*	Time frame	APC2	95%*CI*
Farmers	-1.77*	-1.90, -1.67	2014–2016	-0.79*	-1.30, -0.40	2016–2021	-2.16*	-2.40, -2.03
Housekeepers/House-workers/Unemployed	4.98*	4.35, 5.48	2014–2019	4.00*	2.10, 4.72	2019–2021	7.49*	5.13, 9.34
Students	7.94*	5.20, 11.58	2014–2019	10.82*	4.91, 24.03	2019–2021	1.08	-9.01, 11.40
Retired population	4.62*	3.87, 5.30	2014–2018	2.92*	0.15, 4.22	2018–2021	6.92*	4.98, 9.97
Factory workers	-1.44*	-2.02, -0.98	2014–2018	-2.21*	-4.03, -1.03	2018–2021	-0.41	-2.06, 1.70

Notes: APC, annual percent changes; AAPC, average annual percent changes; *CI*, confidence interval. * represents the $P < 0.05$

Supplement Table 4. The spatiotemporal analysis for TB prefecture-level incidence rates in Chinese mainland

The level of clusters	The number of covered cities	The number of covered cities in PLADs	The period of clusters	*RR*	*LLR*	*P*
1	5	Xinjiang(5)	March 2017–June 2019	8.55	152753.76	< 0.001
2	75	Hubei(17), Hunan(14), Sichuan(13), Guizhou(9), Guangxi(8), Jiangxi(6), Shaanxi(2), Henan(2), Guangdong(1), Hebei(1), Yunnan(1), Chongqing(1)	January 2014–April 2016	1.61	62121.91	< 0.001
3	12	Heilongjiang(12)	January 2014–April 2016	1.89	13188.29	< 0.001
4	6	Anhui(4), Jiangxi(2)	January 2014–April 2016	1.45	1738.5	< 0.001
5	4	Henan(4)	January 2014–April 2016	1.22	670.15	< 0.001
6	2	Henan(2)	September 2014–December 2016	1.45	650.19	< 0.001
7	1	Shaanxi(1)	January 2014–April 2016	1.54	620.43	< 0.001
8	18	Liaoning(13), Jilin(4), Inner Mongolia(1)	January 2014–June 2014	1.33	612.38	< 0.001
9	1	Henan(1)	January 2017–August 2017	1.46	195.88	< 0.001
10	1	Inner Mongolia(1)	March 2016–June 2018	1.36	135.91	< 0.001
11	1	Anhui(1)	November 2018	2.07	111.46	< 0.001
12	1	Inner Mongolia(1)	November 2017–November 2019	1.36	108.99	< 0.001
13	2	Anhui(2)	December 2016–April 2018	1.2	96.16	< 0.001
14	1	Shanxi(1)	January 2014–September 2014	1.34	60.02	< 0.001
15	2	Anhui(2)	January 2014–September 2015	1.1	30.09	< 0.001
16	1	Hebei(1)	January 2019–June 2019	1.22	20.78	< 0.001
17	1	Hebei(1)	January 2015–June 2015	1.33	16.01	0.036

Notes: PLADs, provincial-level administrative divisions; *RR*, relative risk; *LLR*, log-likelihood ratio.

Supplement Table 5. The temporal trends of the proportion for etiologically confirmed cases from 2014 to 2021

Variables	Category	Whole		Period1			Period2		
		AAPC	95%*CI*	Time frame 1	APC1	95%*CI*	Time frame 2	APC2	95%*CI*
Whole	-	9.62*	6.43, 14.61	2014–2016	-5.1	-15.55, 12.42	2016–2021	16.13*	9.66, 33.95
Gender	Male	9.30*	6.32, 13.98	2014–2016	-5.10	-14.95, 11.66	2016–2021	15.65*	9.73, 32.36
	Female	11.40*	8.14, 15.25	2014–2017	0.96	-11.90, 10.89	2017–2021	19.94*	12.58, 37.40
Age group	0–4 years old	25.50*	18.97, 32.48	-	-	-	-	-	-
	5–14 years old	14.16*	11.06, 17.69	2014–2017	4.10	-8.06, 12.81	2017–2021	22.35*	15.87, 37.94
	15–39 years old	9.32*	6.89, 13.16	2014–2016	-5.89	-13.78, 8.14	2016–2021	16.08*	11.65, 29.61
	40–64 years old	8.98*	5.95, 13.70	2014–2016	-5.63	-15.54, 11.16	2016–2021	15.44*	9.46, 32.40
	≥65 years old	10.57*	7.18, 15.77	2014–2016	-3.45	-14.56, 14.47	2016–2021	16.73*	8.54, 35.05
Occupation	Farmers	9.71*	6.45, 14.84	2014–2016	-6.51	-16.93, 11.60	2016–2021	16.96*	10.60, 35.50
	Housekeeping/ Housework/ Unemployment	9.26*	7.05, 12.74	2014–2016	-2.55	-10.05, 10.04	2016–2021	14.38*	10.16, 26.45
	Retired population	8.95*	7.09, 11.73	2014–2016	1.25	-5.37, 11.05	2016–2021	12.20*	7.60, 21.58
	Factory workers	9.74*	7.93, 12.39	2014–2016	-1.81	-7.92, 8.00	2016–2021	14.74*	11.75, 22.38
	Students	13.34*	9.64, 19.27	2014–2016	-4.57	-16.45, 16.21	2016–2021	21.42*	13.89, 43.02
	Others	9.43*	7.36, 12.64	2014–2016	-3.96	-10.85, 7.89	2016–2021	15.29*	11.59, 26.04
PLADs	Beijing	5.17*	3.44, 7.14	2014–2017	-1.05	-8.35, 4.18	2017–2021	10.08*	6.33, 18.81
	Tianjin	7.82*	4.18, 11.62	-	-	-	-	-	-
	Hebei	8.68*	6.22, 12.61	2014–2016	-8.82	-16.57, 5.70	2016–2021	16.58*	12.08, 29.65
	Shanxi	7.43*	4.46, 12.06	2014–2016	-15.91	-24.37, 1.01	2016–2021	18.49*	13.10, 32.40
	Inner Mongolia	12.04*	10.30, 14.46	2014–2016	-6.1	-11.52, 3.19	2016–2021	20.23*	17.24, 25.31
	Heilongjiang	13.46*	7.79, 22.06	2014–2016	-6.83	-23.52, 21.31	2016–2021	22.76*	1.13, 54.60
	Jilin	12.35*	9.22, 16.55	2014–2016	-1.57	-11.70, 11.94	2016–2021	18.45*	13.83, 31.32
	Liaoning	9.85*	7.21, 13.85	2014–2016	-2.8	-11.54, 11.35	2016–2021	15.35*	10.32, 29.27
	Gansu	18.35*	11.20, 29.16	2014–2016	-9.03	-28.22, 25.11	2016–2021	31.49*	16.87, 72.93
	Ningxia	11.97*	10.57, 13.70	2014–2019	15.02	13.35, 19.67	2019–2021	4.70*	-0.44, 10.72
	Qinghai	8.95*	2.51, 19.28	2014–2016	-17.2	-34.86, 16.01	2016–2021	21.60*	6.66, 61.88
	Shaanxi	22.66*	15.37, 30.42	-	-	-	-	-	-
	Xinjiang	16.21*	8.76, 25.07	2014–2017	-7.15	-31.01, 12.53	2017–2021	37.50*	20.68, 83.18

Fujian	2.33*	0.23, 4.26	2014–2018	-3.13	-11.05, 0.64	2018–2021	10.09*	4.01, 20.10
Jiangsu	10.47*	9.16, 12.06	2014–2016	5.09	0.17, 9.96	2016–2021	12.70*	11.25, 17.19
Jiangxi	1.20*	0.25, 2.13	2014–2019	-2.15*	-3.71, -1.07	2019–2021	10.08*	5.02, 14.25
Shandong	12.34*	9.46, 15.63	2014–2017	2.19	-9.24, 10.36	2017–2021	20.61*	14.39, 35.33
Shanghai	9.55*	3.32, 16.22	-	-	-	-	-	-
Zhejiang	8.19*	6.08, 11.20	2014–2016	1.03	-6.33, 11.76	2016–2021	11.19*	2.65, 21.31
Anhui	9.17*	6.13, 13.89	2014–2016	-5.33	-15.29, 11.61	2016–2021	15.57*	9.53, 32.38
Henan	12.63*	9.19, 17.97	2014–2016	-2.52	-13.66, 15.90	2016–2021	19.33*	11.97, 38.48
Hubei	6.52*	5.57, 7.56	2014–2017	-3.48*	-5.96, -0.71	2017–2021	14.70*	12.53, 17.10
Hunan	5.82*	3.86, 8.07	2014–2017	-2.41	-10.88, 3.51	2017–2021	12.44*	8.21, 22.70
Guangdong	15.10*	10.04, 20.43	-	-	-	-	-	-
Guangxi	9.70*	5.16, 16.38	2014–2016	-22.5	34.34, 0.08	2016–2021	26.06*	18.06, 43.78
Hainan	8.11*	5.38, 10.91	-	-	-	-	-	-
Guizhou	13.05*	10.43, 17.22	2014–2016	-2.21	-10.75, 12.70	2016–2021	19.79*	14.82, 34.55
Sichuan	11.78*	9.62, 14.40	2014–2017	-2.42	-13.01, 4.43	2017–2021	23.77*	18.77, 34.07
Xizang	7.12*	3.47, 10.43	2014–2018	-4.28	-18.00, 2.13	2018–2021	24.46*	12.52, 45.31
Yunnan	14.47*	10.03, 19.19	-	-	-	-	-	-
Chongqing	14.90*	10.52, 19.46	-	-	-	-	-	-

Notes: APC, annual percent changes; AAPC, average annual percent changes; *CI* , confidence interval; PLADs, provincial-level administrative divisions. * represents the $P < 0.05$

Supplement Table 6. The *VIF* of five meteorological factors in the model.

Factors	*VIF*
Atemp	1.41
ARH	2.29
AWS	1.07
PRE	1.24
SD	2.00

Notes: *VIF*, variance inflation factor.

Supplement Table 7. The Moran's *I* statistics for incidence rates during 2014–2019

Years	Moran's *I*	Z-value	*P*
2014	0.153	1.755	0.040
2015	0.155	1.778	0.038
2016	0.132	1.558	0.060
2017	0.131	1.563	0.059
2018	0.069	0.965	0.167
2019	0.196	2.152	0.016

Supplement Table 8. The results of LM test

Test	Statistic	*P*
LM-lag test	53.502	<0.001
LM-error test	60.635	<0.001
Robust LM-lag test	15.045	<0.001
Robust LM-error test	22.178	<0.001
Hausman test	43.41	<0.001

Notes: The Hausman test was conducted to select appropriate models (fixed-effect or random-effect). The p-value of the Hausman test was lower than 0.001. Therefore, fixed-effect models were selected. LM tests were then conducted to select the spatial or non-spatial model. The LM tests were conducted to select the spatial or non-spatial model. The p-value of the LM and Robust-LM tests passed the significance level of 5%, indicating the spatial panel model was suitable for the data.

Supplement Table 9. The results of model selection

Test	Statistic	*P*
LR test (SDM vs SLM)	76.13	<0.001
LR test (SDM vs SEM)	61.45	<0.001
Wald (SDM vs SLM)	90.07	<0.001
Wald (SDM vs SEM)	76.03	<0.001

Notes: LR and Wald tests were conducted to select the appropriate spatial model. The LR statistic (SDM vs SLM) was 76.13 ($P < 0.001$), indicating SDM was more suitable for SLM. The

LR statistic (SDM vs SEM) was 61.45 (*P* < 0.001), indicating SDM was more suitable for SEM. The Wald statistic (SDM vs SLM) was 90.07 (*P* < 0.001), indicating SDM was more suitable for SLM. The Wald statistic (SDM vs SEM) was 76.03 (*P* < 0.001), indicating SDM was more suitable for SEM. Therefore, the SDM is more suitable for TB incidence rate. Based on the LM, LR and Wald tests, the fixed-effect SDM was selected for influence factors analysis.

Supplement Table 10. The selection of three SDM model

Test	*AIC*	*BIC*	R^2
cross-section only time	33.07	104.04	0.862
cross-section only individual	-366.51	-295.55	0.019
two-way SDM	-379.24	-308.27	0.013

Notes: SDM model were estimated taking into consideration the fixed effects of cross-section only (individual), year (time) and both cross-section and year (two-way). Based on the *AIC*, *BIC* and R^2 for time, individual and two-way SDM, SDM model with a time fixed effect was selected. *AIC*, Akaike information criterion; *BIC*, Bayesian information criterion; SDM, spatial Durbin model.
